# The Impact of Psychological Distress on the Occupational Well-Being of Sexual and Gender Minorities

**DOI:** 10.3390/healthcare10040699

**Published:** 2022-04-08

**Authors:** Henrique Pereira, Patrícia Silva, Colleen Beatriz

**Affiliations:** 1 Department of Psychology and Education, Faculty of Social and Human Sciences, University of Beira Interior, Pólo IV, 6200-209 Covilhã, Portugal; pg.silva@ubi.pt (P.S.); colleenbeatriz@gmail.com (C.B.); 2 Research Centre in Sports Sciences, Health Sciences and Human Development (CIDESD), 5001-801 Vila Real, Portugal

**Keywords:** psychological distress, occupational well-being, sexual minorities

## Abstract

Background: Discrimination against sexual and gender minorities in occupational settings has been an important topic of research. However, little is known about this impact in Portuguese-speaking people. Methods: 305 Portuguese and Brazilian participants who identified as lesbian, gay, bisexual, transgender, queer, intersex, asexual, and other sexual and gender identities (LGBTQIA+) completed an online survey comprising sociodemographic questions, a set of scales to assess psychological distress, and a set of scales to assess occupational well-being. Results: Participants had higher levels of burnout, depressive symptoms, and anxiety and lower levels of work-related quality of life, engagement, and self-efficacy at work compared to the defined cut-off points for normative populations, with asexual and bisexual people appearing to be the most affected. Significant correlations were found for all variables, and psychological distress was a significant predictor of lower occupational well-being. Conclusions: These findings are useful for understanding the occupational health of LGBTQIA+ people and suggest efforts to improve the climate in the workplace for this population.

## 1. Introduction

Occupational well-being is a broad construct that involves individual and organizational factors that interact and result in the well-being of workers and in relation to their professional engagement [1,2]. This well-being arises through positive feelings about work such as autonomy, belonging, satisfaction, competence, positive peer relationships, personal growth, and work-related quality of life (WRQoL) [1,3,4]. However, negative aspects related to work can reflect on the impairment of occupational well-being, affect the performance of work and increase psychological stress loads and physical and emotional health problems [1,3,4].

Sexual and gender minority individuals, such as lesbian, gay, bisexual, transgender, queer, intersex, asexual and other sexual and gender identities (LGBTQIA+), have historically been targets of discrimination and prejudice in the workplace [5,6,7]. Examples of this include negative comments and behaviors, moral harassment, reduced job opportunities, job offers, and promotions, and stigmatization and violence based on their sexual orientation and/or gender identity, whether by colleagues or business management [8,9,10]. This is particularly relevant since, according to the minority stress model, remaining in an oppressive environment, characterized by heterosexism, discrimination, stereotypes, and prejudice, has negative consequences on the physical and mental health of sexual and gender minority people [11,12].

In fact, LGBTQIA+ people who have experienced prejudice and negative attitudes in the workplace seem to demonstrate higher levels of work-related stress, isolation, and greater psychological distress [13,14,15,16]. Some people also choose to hide their sexual orientation and/or gender identity in the work environment for fear of retaliation [8,17]. These consequences also seem to impact work-related outcomes such as increased levels of absenteeism, turnover, decreased commitment, self-efficacy, motivation, satisfaction, commitment, quality of life, and well-being at work [5,6,18] and, consequently, the levels of productivity, quality, and the results of the organization, itself, can also be affected [5,19]. Evidence also suggests that LGBTQIA+ discrimination is associated with higher levels of unemployment [20,21,22]. Qualitative data from Australia support these quantitative findings, indicating that many transgender workers fear coming out at work, want to avoid work during their transition, and tend to avoid workplaces and stay in higher education institutions and training programs for longer out of fear of workplace difficulties or discrimination [23]. Importantly, longitudinal research also indicates that for transgender employees, job satisfaction is positively associated with gender affirming surgery and supportive coworkers, indicating that a supportive work environment may positively impact the occupational well-being of LGBTQIA+ workers [24].

During the current coronavirus pandemic, the negative aspects experienced by sexual and gender minorities seem to be even more accentuated, increasing the vulnerabilities of a population that already suffered from previous stressors. Studies carried out in Portugal and Brazil point to higher levels of psychological suffering, depression, anxiety, stress, and fears of losing wages or positions among LGBTQIA+ workers during this period [25,26,27], in addition to a worsening in work-related quality of life and occupational well-being [28]. In a qualitative study with 65 Brazilian and Portuguese LGBTQIA+ people, mental health problems were mentioned 78 times by the participants, and more than a quarter of the sample reported problems at work related to the pandemic context and their LGBTQIA+ identity [29].

While a recent study from Canada indicated that LGBTQIA+ individuals with poorer mental health have higher odds of numerous adverse occupational outcomes [30], studies from Portugal and Brazil seem to have only focused on the existence of psychological distress and organizational indicators of LGBTQIA+ people separately thus far. Although the effects of this suffering on occupational well-being have not yet been considered in Portugal and Brazil, assessing this relationship is important, because problems in the organizational context of LGBTQIA+ people are not limited to the increase in psychological distress. They can also impact their perception of work, well-being, and WRQoL, in addition to business results.

We hypothesized that higher levels of psychological distress are associated with lower levels of occupational well-being and that each unique measure of psychological distress (i.e., burnout, depression, and anxiety) is a predictor of each unique measure of occupational well-being (i.e., WRQoL, work engagement, and occupational self-efficacy) among Portuguese and Brazilian sexual and gender minorities. Thus, the objective of this research was to evaluate the association between psychological distress on the occupational well-being of Portuguese and Brazilian sexual and gender minorities. Specifically, we aimed to evaluate both the overall association between psychological distress and occupational well-being as well as the associations between each individual psychological distress variable and each individual occupational health indicator in order to collaborate with the scientific knowledge in this area and enable the identification of measures to recognize and improve organizational, psychosocial, and political interventions in these settings.

## 2. Materials and Methods

### 2.1. Measurement Instruments

*Sociodemographic questionnaire*: Sociodemographic variables included age, gender, sexual orientation, country of residence, professional status, marital status, place of residence, educational attainment, socioeconomic status, nature of the organization they worked for, sector of activity, weekly hours of workload, and shift work.

*Psychological distress*: We used burnout, depression, and anxiety symptoms as measures of psychological distress.

To measure burnout, this study used the Portuguese version of the Burnout Assessment Tool (BAT) [31]. The BAT is a 22-item scale that measures four fundamental symptoms of burnout: exhaustion, mental distance, emotional impairment, and cognitive impairment. Additionally, because burnout is a syndrome that encompasses all four interrelated dimensions [32], we calculated an overall burnout score by taking an overall mean of all 22 items, which were each rated on a five-point Likert scale ranging from 1 (never) to 5 (always). This scale has a Cronbach’s alpha of 0.947, indicating excellent internal reliability.

To measure mental health symptoms, we used the Portuguese version of the Brief Symptom Inventory (BSI-18), specifically the anxiety and depression sub-scales, as these are the most prevalent mental health issues in the general population [33]. The BSI-18 is an 18-item scale that measures depression, anxiety, and somatic symptoms in nonclinical and community populations and has been shown to effectively measure psychological distress and mental health symptoms. For this study, participants self-rated their mental symptoms using 12 items (six for anxiety and six for depression) which were evaluated on a five-point scale ranging from 0 (never) to 4 (always). We then calculated a total mental health symptoms score using a simple mean score, where higher scores reflected more mental health symptoms. Both factors were found to have high internal reliability, with Cronbach’s alpha levels of 0.864 and 0.904 for depression and anxiety, respectively.

*Occupational well-being*: We used WRQoL, work-engagement, and occupational self-efficacy as measures of occupational well-being.

To measure WRQoL, we used the Portuguese version of the Work-Related Quality of Life Scale. This is a 23-item survey that assesses participants’ perception of their WRQoL in their institution or organization [34,35] using a five-point Likert-type scale (1—“Strongly disagree”; 5—“Strongly agree”). The survey includes six psychosocial dimensions: general well-being (feelings of happiness and satisfaction with life), home–work interface (the relationship and balance between personal and professional life), career satisfaction (level of satisfaction with career and work), control at work (level of perceived control in the execution of professional tasks in the work environment), working conditions (related to the working conditions, safety, and resources that the person has in his/her workplace), and stress at work (related to the level of stress that the person perceives related to his/her work) which was reversely coded. For our study, we included a 24th item, “I am satisfied with the overall quality of my working life”, to measure overall perceptions of WRQoL. Internal consistency was excellent (α = 0.92) [34,35].

To measure work engagement, defined as a positive, fulfilling, work-related state of mind characterized by vigor, dedication, and absorption [36], we used the Portuguese version of the Utrecht Work Engagement Scale (UWES) [37]. The Portuguese version consists of nine items, rated on a seven-point Likert-type scale with values ranging from 1 (never) to 7 (always). Examples of items include “I am enthusiastic about my work” and “At my work, I feel strong and vigorous”. We calculated a work-engagement total score using a simple mean score, where higher scores reflected greater work engagement. This scale has a Cronbach’s alpha of 0.94, indicating excellent internal reliability.

To measure occupational self-efficacy, we used the short version of the Occupational Self-Efficacy Scale [38]. This scale uses six items to assesses individuals’ confidence or belief in their ability to cope with difficult tasks or problems within an occupational environment. We translated all items to Portuguese, following existing guidelines for the translation of research instruments [39]. Items were rated using a six-point Likert-type response scale ranging from 1 (not at all true) to 6 (completely true), where higher values reflected greater occupational self-efficacy. Examples of items include: “I can remain calm when facing difficulties in my job because I can rely on my abilities” or “When I am confronted with a problem in my job, I can usually find several solutions”. We calculated an occupational self-efficacy total score using a simple mean score, where higher scores reflected higher levels of self-efficacy. This scale has a Cronbach’s alpha of 0.89, again indicating excellent internal reliability.

Because all of our measures of psychological distress and occupational well-being used previously validated scales (i.e., BSI-18, Work-Related Quality of Life Scale, UWES, and the Occupational Self-Efficacy Scale), and discriminant validity requires other measures to assess the degree to which each measure diverges from a construct conceptually unrelated to it, we did not conduct any discriminant validity calculations in this study.

### 2.2. Procedures

We carried out data collection online from October 2020 to December 2020 using a website created specifically for the purpose of this study. Participation was voluntary and no monetary gratification or compensation was provided. Sample recruitment was conducted among LGBTQIA+ individuals using social networks, LGBTQIA+ organizations, mailing lists, and electronic notifications. Upon receiving the research link, the site provided participants with the research objectives, information for filling out the survey, informed consent, and researchers’ contacts. After sending a total of 1525 notifications, 305 participants agreed to complete the survey and met all inclusion criteria (response rate of 20%). This research met all ethical criteria, including informed consent, anonymity, and confidentiality. To be included in the study sample, participants had to be 18 years of age or older, a Portuguese or Brazilian individual capable of reading Portuguese, and a self-identified LGBTQIA+ individual. This study was approved by the Ethics Committee of the University of Beira Interior (Portugal) (CE-UBI-PJ-2020-088).

### 2.3. Data Analysis

Descriptive statistics, including means, standard deviations, frequencies, and percentages, were calculated to describe the sample. The Kolmogorov–Smirnov test was used to evaluate and confirm the normality of the data. ANOVA tests were used to assess differences between comparison groups, Pearson’s correlation coefficients were calculated to assess the association between variables, and simple linear logistic regression analyses were used to assess the predictive power of psychological distress on occupational well-being. To measure internal consistency, we used Cronbach’s alpha. We used Bonferroni correction tests to avoid Type I errors and measured multicollinearity using the variance inflation factor (VIF = 1), which indicated that the variables were not correlated. All statistical procedures were conducted using the Statistical Package for the Social Sciences (SPSS version 27, IBM Corporation, Armonk, NY, USA).

## 3. Results

A total of 305 Portuguese and Brazilian LGBTQIA+ individuals between 18 and 67 years of age (*M_age_* = 31.97; *SD* = 11.85) participated in the study. The majority (54.7%) identified as male, were single (75.3%), had a university education (72.6%), resided in urban settings (86%), and reported belonging to the middle socioeconomic status (50.5%). Regarding their occupational status, the majority indicated that they were employed (49.2%), worked for public organizations (50%), worked in the tertiary sector (94.3%), and did not do shift work (79.7%). Table 1 shows the sociodemographic characteristics in more detail.

Overall, psychological distress scores were higher than expected when compared to community samples, where the majority of participants are straight, indicating that sexual and gender minority participants may be at higher risk for developing mental health problems. More specifically, the mean level of burnout in the community samples was 2.37 (*SD* = 0.60) [40], whereas in our sample, a mean score of 2.68 (*SD* = 0.69) was obtained. The mean level of depression symptoms in the community samples was 0.94 (*SD* = 0.84) [40], whereas in our sample a mean score of 1.43 (*SD* = 0.97) was obtained. The mean level of anxiety symptoms in the community samples was 0.93 (*SD* = 0.73) [40], whereas a mean score of 1.39 (*SD* = 0.85) was found in our sample. The same trend was observed for occupational well-being indicators: mean levels of work-related quality of life were lower in our sample (3.20; *SD* = 1.03) than in samples where most participants were straight (3.40; *SD* = 0.96) [28], work engagement mean scores were lower in our sample (4.12; *SD* = 1.43) than in samples where the majority of participants were straight (4.65; *SD* = 1.30), and occupational self-efficacy mean scores in our study were lower (3.31; *SD* = 0.89) than in samples where the majority of participants were straight (3.66; *SD* = 0.81) [41].

In addition, we analyzed differences in psychological distress variables (i.e., burnout, depression, and anxiety) and occupational well-being indicators (i.e., WRQoL, work engagement, and occupational self-efficacy) by sexual orientation. The results show statistically significant differences (*p* < 0.05) for depression and anxiety symptoms, indicating that self-identified asexual and bisexual participants presented higher psychological distress scores than gay/lesbian and pansexual participants. Asexual and bisexual participants also scored lower on all occupational well-being indicators, although these differences were not statistically significant (see Table 2 for further details and Table S1 for differences uncovering specific differences between the four group means).

A correlation matrix was created to assess the levels of association between psychological distress variables (i.e., burnout, depression, and anxiety) and occupational well-being indicators (i.e., WRQoL, work engagement, and occupational self-efficacy). As shown in Table 3, psychological distress variables were strongly, negatively, and significantly (*p* < 0.001) correlated with occupational well-being variables. Age was negatively correlated with burnout, depression, and anxiety and positively correlated with occupational self-efficacy.

Finally, we conducted nine simple logistic regressions for psychological distress variables predicting each occupational well-being indicator. As displayed in Table 4, all models were significant (*p* < 0.001), and burnout was a negative and strong predictor that explained 27% of the variability in low work-related quality of life, 47% of the variability in low work engagement, and 33% of the variability in lower occupational self-efficacy. Depression symptoms were a negative and strong predictor that explained 14% of the variability in low WRQoL, 26% of the variability in low work engagement, and 20% of the variability in low occupational self-efficacy. Anxiety symptoms were a negative and strong predictor that explained 12% of the variability in low WRQoL, 19% of the variability in low work engagement, and 12% of the variability in low occupational self-efficacy.

## 4. Discussion

The present study sought to analyze the impacts of psychological distress on the occupational well-being of sexual and gender minorities living in Portugal and Brazil. Our participants had higher levels of burnout, depressive symptoms, and anxiety symptoms than previously found in community samples, although it was not possible to estimate the effect sizes. In addition, our sample had lower levels of WRQoL, work engagement, and occupational self-efficacy compared to previous study samples in which most participants were heterosexual. In all study variables, asexual and bisexual people seemed to be the most affected. We found a high and significant negative correlation between the three variables of psychological distress, namely, burnout, depression, and anxiety symptoms, and the variables related to occupational well-being. Finally, consistent with our hypothesis, we found that all psychological distress variables were significant predictors of lower occupational well-being indicators. These results raise important questions for understanding the occupational health of LGBTQIA+ people both in and beyond the context of crisis.

Our participants had higher-than-expected scores for all psychological distress variables and lower scores for occupational well-being variables. This is consistent with existing research that indicates that people who suffer from social stigma, such as being a belonging of a sexual and/or gender minority, seem to be the most vulnerable to external and internal stressors in the workplace such as formal discrimination, interpersonal discrimination, stigma consciousness, internalized heterosexism, concealment, and social isolation [14,35,36]. This workplace stress is associated with increased psychological stress and health problems in addition to influencing workplace outcomes, resulting in fewer opportunities and promotions for these people [14,42,43,44]. Furthermore, organizational results are affected by low productivity and high staff turnover due to the consequences of prejudice and discrimination, which leads to a reduction in profits [5,45].

Because data collection was carried out during the COVID-19 pandemic, it is important to interpret our results through this context. The very unpredictable situation of the pandemic may have exacerbated pre-existing vulnerabilities in our sample [46,47,48], and these results point to the need to work on disparities regarding sexual orientation and gender identity in the organizational context. In particular, it is important to take the pandemic situation into account, as new work structures and work recovery processes are still slow and uncertain [49,50].

Bisexual and asexual participants scored the highest for the variables of psychological distress, with a significant difference for depression and anxiety. Consequently, they, along with pansexual participants, were also the ones who had the lowest scores for occupational well-being variables such as WRQoL, work engagement, and occupational self-efficacy. These results were expected if we consider the monosexist and binomial assumptions that people should be attracted to one specific gender [51,52], often putting people who are not in that position in internal conflicts [53,54], being pressured to assume a sexual script that does not reflect their true sexual experience and identity [55,56].

In fact, studies show that bisexual and pansexual people tend to be more likely to present negative outcomes related to their biopsychosocial health than monosexual people (i.e., heterosexuals, gays, and lesbians) [57,58,59]. This is because they tend to suffer discrimination on both the heterosexual and homosexual fronts [60,61,62] and are often stereotyped as being afraid to assume their homosexual identities [63,64,65] or as being promiscuous and unfaithful [66,67]. Asexual people go through situations such as those of bisexuals and pansexuals because they do not meet a normative monosexual and binomial position. Because asexual people do not fit into the sex-normative society that is dominated by certain forms of sexuality [68,69], they may suffer stigma. For example, asexuality is often viewed as something wrong, such as a disorder, or treated with disbelief and rejection [61,62]. In this sense, the discrimination suffered by bisexuals, pansexuals, and asexuals can impact their own acceptance and/or concealment of their identity [54,70,71]. This discrimination is also associated with increased depression and anxiety symptomology [55,72,73,74,75] and worse mental health [76,77], general well-being [72,78], and work experiences [28,69,79].

Finally, our most relevant results sought to assess whether psychological distress acted as a determining factor for lower occupational well-being in the sample. All models were negative and significant. These results corroborate previous studies and others conducted during the COVID-19 pandemic that found a negative relationship between psychological distress and the occupational well-being of LGBTQIA+ people [28,29,79,80,81]. Burnout seems to have the greatest impact on occupational well-being, followed by depression and anxiety, consecutively. In fact, burnout has already been identified as a significant predictor of mental health symptoms; that is, emotional exhaustion is related to the worsening of mental health symptoms and consequent worsening of occupational well-being [82,83,84]. A recent study found that when burnout was considered as a mediator, the occupational well-being variable WRQoL did not have a significant effect on reducing mental health symptoms [85]. However, where WRQoL was better, there was a decrease in burnout and, consequently, a reduction in symptoms of anxiety, depression, and somatization. This conceptual relationship may explain why burnout appears in our study as a major determinant of occupational well-being.

### 4.1. Implications

From a practical point of view, our results may have implications for the intervention in the occupational health field, corroborating the understanding of the occupational well-being of sexual and gender minorities and supporting the construction of care proposals for this population [86], mainly in the context of crisis, such as the COVID-19 pandemic [20]. Furthermore, we point to the need for the involvement of society and the political system for the promotion and maintenance of labor rights for the LGBTQIA+ community [87]. At the corporate level, the results suggest that organizational efforts aimed at improving the biopsychosocial health of LGBTQIA+ people should focus on reducing psychological distress, especially symptoms of burnout, depression, and anxiety that directly affect occupational well-being. These efforts may also benefit from the promotion of WRQoL, occupational engagement, and self-efficacy, which were positively correlated in the multifaceted construct of occupational well-being in our study.

In this sense, organizational measures, with a focus on diversity management, can help to minimize psychological stress and improve occupational well-being. Examples of these measures may include: (1) making work more egalitarian and providing opportunities for diversified work teams; (2) taking steps to minimize prejudice and discrimination in the workplace; (3) adjusting labor policies and norms with inclusive benefits and opportunities; (4) ensuring a reliable working climate among employees, favoring acceptance, and reducing fears regarding sexual identity; (5) provide domestic partner benefits; (6) engaging unions and employers to strengthen an inclusive workplace [45,88,89]. In fact, companies that implement measures to support sexual and gender minorities tend to have lower levels of discrimination, which allows LGBTQIA+ people to tend to feel better in the workplace which, in turn, tends towards greater productivity and, ultimately, better organizational results [90,91,92,93].

Additionally, it is vital that organizations include LGBTQIA+ workers in decision-making processes regarding mental health and occupational well-being promotion, as employees and employers often have differing perceptions of what measures are adequate. School-based evidence from Vietnam, for example, indicated that while 95.4% of teachers and school administrators believed that appropriate measures were in place to address anti-LGBTQIA+ violence and discrimination, only 14.6% of students agreed [94]. While similar research has not been conducted in the workplace to our knowledge, this study illustrates the disparity between decision makers and LGBTQIA+ stakeholders and highlights the need for LGBTQIA+ voices decision-making processes.

### 4.2. Limitations and Future Directions

However, this study has its limitations. The first is related to the profile of the sample, taken for convenience, which was formed mostly by single people with at least a university education, who resided in urban areas and with an average socioeconomic status, which interferes with the representativeness of the population. In addition, approximately 94% of the participants worked in the tertiary sector and without shifts, which also limits the generalizability of results to other sectors. Moreover, in this context, the questionnaire was made available online and in a self-application format, indicating the possibility of selection bias, since only people with internet access could respond to the survey. In this sense, future studies could benefit from larger, more differentiated, and representative samples, which allow for better generalization of results. While 73% of the sample had a university education, only 51% deemed themselves as having a mid-level socioeconomic status, which may suggest that many were underemployed or held positions below their expectations, which might account for some of the negativity associated with work other than discrimination. Moreover, our data relied mainly on sexual minority stress theory, and since there was not a measure for discrimination or stigmatization in this study, the research cannot directly address the effects of those factors on the outcome variables. Future research should address these limitations by including other possible explanations and a measure of sexual stigma.

The transversality of the study is also a limitation. The COVID-19 pandemic already appears to be a threat to vulnerable populations [46,47,48] and may have influenced the responses of our participants. Longitudinal studies can assess the change in these aspects over time to improve the understanding of the relationship between psychological stress and occupational well-being during and beyond the COVID-19 pandemic. Finally, as well-being is a complex construct, it could be useful to add additional measures that focus on the assessment of the biopsychosocial health of participants in life contexts other than the professional [14], in addition to evaluating the experience of participants in diversity management policies in the companies where they work. These answers could bring new interpretations to the results and help in the verification of predictive and preventive factors of occupational well-being for this population.

## 5. Conclusions

This study contributes to the understanding of sexual and gender minority occupational health through the understanding of issues related to psychological distress and the impact of this stress on organizational well-being. The results corroborate previous studies and others similarly developed during the COVID-19 pandemic, which also found that sexual and gender minority people had worse outcomes related to psychological stress and occupational well-being in this period. These findings suggest that organizations and professionals in the occupational health area should be aware that certain groups of workers may be more vulnerable to situations of discrimination, prejudice, and other occupational risks, as in the case of sexual and gender minority populations. Providing a more inclusive and comfortable work environment, with a well-designed diversity management policy that focuses on improving the organizational context in the long term can have benefits for the biopsychosocial health of workers in all their diversity and, consequently, in the delivery of individual and organizational benefits.

## Figures and Tables

**Table 1:** Sociodemographic characteristics (*M_age_* = 31.97; *SD* = 11.85).

Variable	Category	*n*	%
Gender	Male	167	54.7
	Female	133	43.7
	Other	5	1.6
Sexual Orientation	Gay/lesbian	146	47.9
	Bisexual	140	45.9
	Pansexual	13	4.3
	Asexual	6	1.9
Country of Residence	Portugal	185	60.7
	Brazil	120	39.3
Professional Status	Student	94	30.8
	Employed	165	54.1
	Self-employed	29	9.5
	Unemployed	11	3.6
	Retired	6	2.0
Educational Attainment	Middle school	6	2.0
	High school	77	25.2
	Bachelor’s degree	102	33.4
	Master’s degree	79	25.9
	Doctorate/PhD	41	13.5
Socioeconomic Status	Very low	19	6.2
	Low	91	29.8
	Middle	154	50.5
	High	35	11.5
	Very high	6	2.0
Marital Status	Single	230	75.4
	Same-sex de facto union	35	11.5
	Same-sex marriage	25	8.2
	Divorced/separated	13	4.3
	Widowed	2	0.6
Place of Residence	Small rural area	27	8.9
	Large rural area	16	5.2
	Small urban area	116	38.0
	Large urban area	146	47.9
Shift Work	Yes	62	20.3
	No	243	79.7
Nature of the Organization	Public	153	50.1
	Private	112	36.7
	Other	40	13.2
Sector of Activity	Primary	11	3.6
	Secondary	6	2.0
	Tertiary	288	94.4

**Table 2:** Differences in psychological distress variables and occupational well-being indicators by sexual orientation.

Variable	Sexual Orientation	Mean	SD	Minimum	Maximum	F	*p*
Burnout	Asexual	3.32	1.23	1.77	4.36		
	Bisexual	2.74	0.63	1.00	4.77		
	Gay/Lesbian	2.61	0.73	1.09	5.00		
	Pansexual	2.46	0.43	2.05	3.09		
	Total	2.68	0.69	1.00	5.00	2.037	0.110
Depression	Asexual	2.87	0.80	1.67	3.33		
	Bisexual	1.63	0.93	0.00	4.00		
	Gay/Lesbian	1.19	0.91	0.00	3.50		
	Pansexual	1.29	1.23	0.00	3.83		
	Total	1.43	0.97	0.00	4.00	6.424	0.000 **
Anxiety	Asexual	2.08	0.61	1.50	2.83		
	Bisexual	1.53	0.86	0.00	4.00		
	Gay/Lesbian	1.30	0.81	0.00	3.67		
	Pansexual	0.62	0.66	0.00	1.67		
	Total	1.39	0.85	0.00	4.00	4.304	0.006 *
Work-Related Quality of Life	Asexual	2.50	1.73	1.00	4.00		
	Bisexual	3.18	0.96	1.00	5.00		
	Gay/Lesbian	3.23	1.07	1.00	5.00		
	Pansexual	3.37	0.91	2.00	5.00		
	Total	3.20	1.03	1.00	5.00	0.728	0.537
Work Engagement	Asexual	3.30	2.39	1.00	5.89		
	Bisexual	4.04	1.33	1.33	7.00		
	Gay/Lesbian	4.18	1.50	1.00	7.00		
	Pansexual	4.57	0.95	3.00	6.11		
	Total	4.12	1.43	1.00	7.00	0.832	0.478
Occupational Self-Efficacy	Asexual	2.45	1.41	1.00	4.17		
	Bisexual	3.25	0.89	1.33	5.00		
	Gay/Lesbian	3.42	0.87	1.33	5.00		
	Pansexual	3.14	0.76	2.17	4.33		
	Total	3.31	0.89	1.00	5.00	1.955	0.122

* *p* < 0.05; ** *p* < 0.001.

**Table 3:** Correlation matrix.

Variable	1	2	3	4	5	6	7
1-Age	1						
2-Burnout	−0.157 *	1					
3-Depression	−0.146 *	0.714 **	1				
4-Anxiety	−0.189 *	0.701 **	0.752 **	1			
5-WRQoL	0.097	−0.518 **	−0.378 **	−0.339 **	1		
6-Work engagement	0.087	−0.680 **	−0.513 **	−0.437 **	0.641 **	1	
7-Occupational self-efficacy	0.254 *	−0.575 **	−0.450 **	−0.343 **	0.478 **	0.620 **	1

* *p* < 0.05; ** *p* < 0.001.

**Table 4:** Simple logistic regressions for psychological distress variables predicting occupational well-being indicators.

*Predictor*	*Occupational Well-Being Indicator*	*B*	*SE*	*β*	*R* ^2^	*F*
Burnout	WRQoL	−0.767	0.092	−0.518 **	0.269	69.064 **
	Work engagement	−1.388	0.110	−0.680 **	0.468	159.148 **
	Occupational self-efficacy	−0.731	0.076	−0.575 **	0.331	91.441 **
Depression	WRQoL	−0.393	0.072	−0.378 **	0.143	29.930 **
	Work engagement	−0.739	0.093	−0.513 **	0.263	62.886 **
	Occupational self-efficacy	−0.407	0.061	−0.450 **	0.203	44.778 **
Anxiety	WRQoL	−0.404	0.084	−0.339 **	0.115	23.181 **
	Work engagement	−0.732	0.114	−0.437 **	0.191	41.550 **
	Occupational self-efficacy	−0.359	0.074	−0.343 **	0.118	23.452 **

** *p* < 0.001.

## Data Availability

The data presented in this study are available upon request.

## Supplementary Materials

The following supporting information can be downloaded at: https://www.mdpi.com/article/10.3390/healthcare10040699/s1, Table S1: Multiple Comparisons Tukey HSD.
