# Supplementary material for: The Impact of Psychological Distress on the Occupational Well-Being of Sexual and Gender Minorities
Source: Healthcare (Basel). 2022 Apr 8;10(4):699. doi: 10.3390/healthcare10040699 (PMC9026495; doi:10.3390/healthcare10040699)
Supplement: Supplementary file 1 [file healthcare-10-00699-s001.zip › healthcare-1635875-Supplementary Material.pdf]

Table S1 – Multiple Comparisons Tukey HSD

Variable	(I) Sexual Orientation	(J) Sexual Orientation	Mean Difference (I-J)	Standard Error	Sig.
Depression Symptoms	Asexual	Bisexual	1.40476	0.64021	0.129
		Gay or Lesbian	1.89773*	0.63955	0.018
		Pansexual	1.87500	0.76607	0.072
	Bisexual	Asexual	-1.40476	0.64021	0.129
		Gay or Lesbian	0.49297	0.19082	0.051
		Pansexual	0.47024	0.46287	0.740
	Gay or Lesbian	Asexual	-1.89773*	0.63955	0.018
		Bisexual	-0.49297	0.19082	0.051
		Pansexual	-0.02273	0.46196	1.000
	Pansexual	Asexual	-1.87500	0.76607	0.072
		Bisexual	-0.47024	0.46287	0.740
		Gay or Lesbian	0.02273	0.46196	1.000
Anxiety Symptoms	Asexual	Bisexual	1.63855*	0.54413	0.016
		Gay or Lesbian	1.81818*	0.54342	0.005
		Pansexual	3.00000*	0.65092	0.000
	Bisexual	Asexual	-1.63855*	0.54413	0.016
		Gay or Lesbian	0.17963	0.16264	0.687
		Pansexual	1.36145*	0.39351	0.004
	Gay or Lesbian	Asexual	-1.81818*	0.54342	0.005
		Bisexual	-0.17963	0.16264	0.687
		Pansexual	1.18182*	0.39252	0.016
	Pansexual	Asexual	-3.00000*	0.65092	0.000
		Bisexual	-1.36145*	0.39351	0.004
		Gay or Lesbian	-1.18182*	0.39252	0.016
Burnout	Asexual	Bisexual	0.58031	0.35405	0.359
		Gay or Lesbian	0.71955	0.35363	0.179
		Pansexual	0.86364	0.42409	0.178
	Bisexual	Asexual	-0.58031	0.35405	0.359
		Gay or Lesbian	0.13925	0.10299	0.531
		Pansexual	0.28333	0.25573	0.685
	Gay or Lesbian	Asexual	-0.71955	0.35363	0.179
		Bisexual	-0.13925	0.10299	0.531
		Pansexual	0.14408	0.25516	0.942
	Pansexual	Asexual	-0.86364	0.42409	0.178
		Bisexual	-0.28333	0.25573	0.685
		Gay or Lesbian	-0.14408	0.25516	0.942
Work Engagement	Asexual	Bisexual	-0.74051	0.73463	0.745
		Gay or Lesbian	-0.87545	0.73261	0.631
		Pansexual	-1.27431	0.87857	0.470
	Bisexual	Asexual	0.74051	0.73463	0.745
		Gay or Lesbian	-0.13493	0.21733	0.925
		Pansexual	-0.53379	0.53141	0.747
	Gay or Lesbian	Asexual	0.87545	0.73261	0.631
		Bisexual	0.13493	0.21733	0.925
		Pansexual	-0.39886	0.52861	0.875
	Pansexual	Asexual	1.27431	0.87857	0.470
		Bisexual	0.53379	0.53141	0.747
		Gay or Lesbian	0.39886	0.52861	0.875
Occupational Self-Efficacy	Asexual	Bisexual	-0.79428	0.45551	0.304
		Gay or Lesbian	-0.96884	0.45449	0.147
		Pansexual	-0.68750	0.54491	0.588
	Bisexual	Asexual	0.79428	0.45551	0.304
		Gay or Lesbian	-0.17456	0.13471	0.567
		Pansexual	0.10678	0.32942	0.988
	Gay or Lesbian	Asexual	0.96884	0.45449	0.147
		Bisexual	0.17456	0.13471	0.567
		Pansexual	0.28134	0.32800	0.827
	Pansexual	Asexual	0.68750	0.54491	0.588
		Bisexual	-0.10678	0.32942	0.988
		Gay or Lesbian	-0.28134	0.32800	0.827
Work-related Quality of Life	Asexual	Bisexual	-0.68605	0.52989	0.567
		Gay or Lesbian	-0.73656	0.52901	0.506
		Pansexual	-0.87500	0.63440	0.514

	Bisexual	Asexual	0.68605	0.52989	0.567
		Gay or Lesbian	-0.05051	0.15498	0.988
		Pansexual	-0.18895	0.38293	0.960
	Gay or Lesbian	Asexual	0.73656	0.52901	0.506
		Bisexual	0.05051	0.15498	0.988
		Pansexual	-0.13844	0.38170	0.984
	Pansexual	Asexual	0.87500	0.63440	0.514
		Bisexual	0.18895	0.38293	0.960
		Gay or Lesbian	0.13844	0.38170	0.984

*$p<0.05$
